# Efficacy of Hearing Aids in Patients with Hearing Difficulties in Noise: Focus on Hidden Hearing Loss

**DOI:** 10.3390/jcm14020360

**Published:** 2025-01-09

**Authors:** Marc Boulet, Marine Veleur, Julie Chédeau, Jérôme Lefeuvre, Gérald Fain, Antoine Paul, Mihaela Alexandru, Jérôme Nevoux

**Affiliations:** 1 Assistance Publique–Hôpitaux de Paris, Hôpital Bicêtre, Service d’Oto-Rhino-Laryngologie, 78 Rue du Général Leclerc, 94270 Le Kremlin-Bicêtre, France; m.boulet@audition-marcboulet.fr (M.B.); marine.veleur@gmail.com (M.V.); j.chedeau@audition-marcboulet.fr (J.C.); j.lefeuvre@audition-lefeuvre.fr (J.L.); geraldfain1@gmail.com (G.F.); antoine.paul@aphp.fr (A.P.); mihaela.dana.alexandru@gmail.com (M.A.); 2 Audition Marc Boulet, 91200 Athis-Mons, France; 3 Audition Lefeuvre, 75012 Paris, France; 4 Faculté de Médecine, Université Paris-Saclay, 94275 Le Kremlin-Bicêtre, France

**Keywords:** hidden hearing loss, hearing aids, speech intelligibility in noise, tinnitus

## Abstract

**Objectives:** Hearing aids (HAs) have been used for standard high-frequency hearing loss and tinnitus, but their effects on speech intelligibility in noise (SIN) in people with normal hearing, including hidden hearing loss (HHL), have been little explored. **Methods:** We included in a prospective cohort study patients who experience poor SIN and have normal pure tone average in quiet conditions or slight HL. We used open-fit HAs. The FraMatrix test was used to explore SIN. The benefit of HAs in daily situations and for tinnitus was evaluated. **Results:** Forty patients were explored, including 12 with HHL. FraMatrix and quality of life improved significantly (*p* < 0.001) with HAs, especially in those with HHL (2.0 dB SNR of speech recognition threshold improvement). HAs also effectively suppress tinnitus. Participants used HAs 9.9 h per day. **Conclusions:** This study shows an improvement of SIN with HAs in patients with slight HL but more importantly in patients with HHL.

## 1. Introduction

Hearing loss is usually defined and diagnosed by means of a pure tone audiogram in quiet situations. Discrepancies are sometimes observed between the patients’ complaints and the slight or zero threshold shift revealed by the usual hearing tests. Such people may be unable to understand speech in a noisy environment [1]. This is known as hidden hearing loss (HHL) or supraliminal hearing disorder [1,2]. The World Health Organization defines HHL as “the condition in which an individual experiences common symptoms associated with noise-related auditory damage, such as difficulty in hearing in noise, and that is undetectable on pure-tone audiometry” [3]. Several peripherals (synaptopathy) to more central (central auditory processing disorder or CAPD) disorders could generate this clinical presentation. To better understand these manifestations in humans, animal models have been developed, including synaptopathy and neuropathy [4]. The difference between synaptopathy and neuropathy is the absence of speech audiometry distortion and normal ABR waves in synaptopathy [4].

In synaptopathy, the affected neurons are silenced by the loss of synaptic connections between inner hair cells (IHCs) and auditory nerve fibers (ANFs), even if it takes months to years for the loss to be reflected in the spiral ganglion [1]. Synapse disconnection first affects low-spontaneous-rate neurons, which are responsible for sensitivity in noisy environments [1,5]. Audiometric thresholds may remain unaffected by diffuse synaptopathy, even if patients experience significant perceptual consequences such as impaired intelligibility of speech in background noise or tinnitus [5].

This clinical presentation with hearing difficulties in noise could also be related to CAPD. CAPD is often described in children but can affect adults of all ages [6]. It has been estimated that CAPD affects 2–5% of the school-aged population, or approximately half of all children with learning disorders, and it can affect up to 76% of elderly adults depending on the criteria used for diagnosis [7]. It includes several characteristics, such as difficulty in understanding language in noisy backgrounds. Patients present reading, spelling, and learning difficulties, poor attention span and being easily distracted, and difficulty in localizing a sound among others [8].

Similarly, aging is often accompanied by listening difficulties in noisy situations, even when classical audiometry reveals a normal hearing threshold [9]. The U.S. Census Bureau (1981) defined middle-aged persons as those of ages 45–64 years. Moreover, age-related hearing loss (ARHL) can begin as early as the fourth decade of age [10]. Thus, we can expect hearing degradation in the middle-aged population related to ARHL. ARHL is a progressive, bilateral, and symmetrical age-related sensorineural HL involving mostly the high frequencies. It is a multifactorial disorder including biological age, gender, ethnicity, environment (noise exposure, drugs), lifestyle (smoking), health comorbidities (hypertension, diabetes), and genetic predisposition [11].

HHL is a perceptual difficulty in hearing that cannot be explained by audiometric results. The tonal and speech audiometry in silence is normal, with a pure tone average threshold < 20 dB HL and speech recognition threshold obtained for 10 dB HL respectively [12]. Patients with HHL complained of hearing difficulties in noise but not in quiet situations. Specific tests for HLL, such as speech in noise tests, have been suggested to highlight these hearing losses [13], but to date, there is no dedicated test to diagnose HHL. CAPD diagnosis is also difficult and includes behavioral and/or electrophysiologic audiologic tests [14].

There is yet no treatment for HHL, but research employs, in models of peripheral insult, strategies to repair or regenerate lost synapses between IHCs and ANFs [15,16]. Since hearing aids (HAs) are effective for sensorineural hearing loss with an auditory threshold shift [17] and for tinnitus [18], we investigated their use in patients with hearing difficulties in noise, especially HHL. Only two studies report hearing aid outcomes in these problems [19,20]. Roup et al. focused on the effect of HAs in adults with substantial difficulty in understanding speech in complex listening situations despite normal pure tone sensitivity in quiet conditions with criteria of CAPD [19]. Singh et al. reported a smaller cohort and mostly studied hearing handicap, motivation, and attitudes toward HA based on questionnaires [20]. The treatment for CAPD is based on three areas, namely environmental modifications, compensatory strategies/central resources training, and direct skills remediation [14].

As specific fitting is expected for this hearing impairment, the use of real-ear measurement (REM) appears to be critical. Indeed, REM tests explore the electroacoustic characteristics of HAs under real conditions by measuring the real-ear output of the HA by placing a probe-tube microphone at the tip of the HA in the ear canal. This is the only objective measure of hearing aid amplification [21]. It guarantees that HAs are open-fit and measures their real amplification.

### Objectives

The aim of this study was to investigate the effects of a conventional type of hearing aid on patients with hearing in noise difficulties, especially HHL, and for some with tinnitus.

## 2. Material and Methods

The study was approved by the SFORL Ethics Committee (9 March 2022) and the Commission Nationale de l’Informatique et des Libertés (CNIL no. 1909577v0). All procedures were in accordance with the Declaration of Helsinki and Good Clinical Practices [22]. All participants provided written informed consent. We followed the STROBE reporting guidelines.

Patients of all ages were included, with normal hearing or slight HL in quiet situations and who had trouble understanding speech in complex listening environments. Patients included had no history of ear trouble or neurologic disorders, no conditions related to auditory processing disorders, and normal otoscopic findings. No patient had previously used HAs.

Data analysis was performed for all patients and separately for patients under 45 years to limit the effect of age on HL and for patients with normal hearing, the so-called HHL. To confirm hearing function in quiet conditions, pure tone audiometry from 250 to 8000 Hz was performed in both unaided ears. We used the World Health Organization’s definition of normal hearing to include patients [23]. Thus, only participants with a pure tone average (PTA) threshold (calculated for 0.5, 1, 2, and 4 kHz) < 20 dB HL on both sides were included. Patients presenting all single thresholds from 250 to 8000 Hz < 20 dB were diagnosed as having HHL.

A Madsen^®^ A450 Astera2 audiometer (Natus^®^, Pleasanton, CA, USA) was used to test air conduction with HDA 300 headphones (Sennheiser^®^ electronic, Wedemark, Germany) and bone conduction threshold with bone conduction headphones (NB71). Speech audiometry was performed in free field and with headphones, in both quiet (using Fournier disyllabic words) and noisy situations (using the French Matrix Test—FraMatrix [24]).

The automatic speech recognition threshold 50% (SRT) search function was used with FraMatrix [24] to determine the maximum signal-to-noise ratio (SNR) possible to obtain SRT. The procedure is adaptive, with noise being set at a 65 dB sound pressure level (SPL). Initially, the signal (speech) level is set at a 65 dB SPL (SNR = 0 dB), then it varies. The test was performed during the first visit without HAs to confirm speech-in-noise disorder and at 6 months, with and without HAs. The average SRT is a −6.0 dB SNR (with a standard deviation of a 0.6 dB SNR across listeners) in normal hearing patients. The standard deviation is only 0.1 dB. The within-subject variability is 0.4 dB. The set-up for this test is simple using the Madsen^®^ A450 Astera2 audiometer and one speaker RCA Siare Acoustique^®^ Alpha 22 HR (Siare Acoustique^®^, Rives-du-Loir-en-Anjou, France) in free field [24].

Tympanometry was performed using Titan (Interacoustics^®^, Middelfart, Denmark), with a probe-tone frequency of 226 Hz.

Participants were fitted with conventional HAs as receiver-in-the-canal (RIC) Audeo M-90 (R or RT) (R for rechargeable and RT for rechargeable with coil) Phonak^®^ (Sonova France SAS, Bron, France) using standard power receivers with an open-fit coupling (open dome). The automatic noise reducer and the automatic directional microphone were activated and used to fit the HA. The HA includes 20 frequency channels. But the dynamic range compression was used only over 80 dB to decrease sound discomfort.

REM tests used to check the fitting of HAs were performed in three different conditions, namely without HAs, with HAs off, and with HAs on. We evaluated REIG (real-ear insertion gain), which corresponds to the gain with the HA off and in the external ear canal. We used the Aurical^©^ FreeFit (Natus Medical^©^, Taastrup, Denmark) and measured gain at five frequencies (1.5, 2, 3, 4, and 6 kHz). The National Acoustics Laboratory’s NAL-NL2 fitting formula was used to determine target gain levels [25].

We explored the efficacy of HAs in HHL patients, both in the total cohort and in a subgroup of patient under 45 years old. We compared with and without HA hearing test results, especially the speech audiogram in quiet and noisy conditions.

We analyzed quality of life (QOL) before and 6 months after the fitting of HAs, with the Abbreviated Profile of Hearing Aid Benefit (APHAB) questionnaire to determine the benefit of HAs in everyday situations [26].

The efficacy of HAs on tinnitus was also explored, as was the impact of tinnitus on QOL, using the Tinnitus Handicap Inventory (THI) score and visual analog scales (VASs) for loudness (VAS-L) and for annoyance (VAS-A), with and without HAs [27].

Statistical analyses, figures, and tables were prepared using Prism 8 software (GraphPad Software, San Diego, CA, USA). The outcomes were described using the mean value (±standard deviation) and [minimum − maximum]. The level of statistical significance was set at 0.05. Correlations were made using a linear regression for two values and an ANOVA for multiple comparisons.

## 3. Results

Forty patients (20 women, 20 men) with a mean age of 53 ± 12.7 [15; 78] (Table 1) were included. Tympanometry was type A in all patients, assuming good middle ear function. The whole cohort and two subgroups were explored, with (1) 10 patients under 45 years old (five women, five men) with a mean age of 36 ± 8.9 [15; 45] and (2) 12 patients with HHL (eight women, four men) with a mean age of 47.8 ± 12.8 (Table 1).

**Figure 1:**
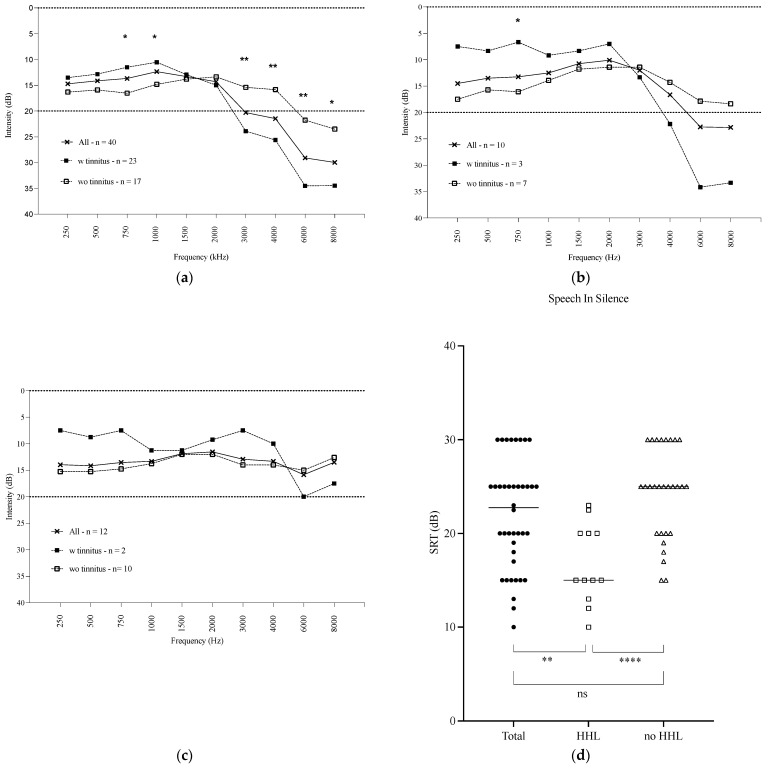
Results of audiometric test in silence. (**a**) Standard frequency pure tone audiometry for the whole cohort (n = 40), subjects with tinnitus (n = 23), and subjects without tinnitus (n = 17); (**b**) standard frequency pure tone audiometry for the younger subgroup (<45 years old) (n = 10), subjects with tinnitus (n = 3), and subjects without tinnitus (n = 7); (**c**) standard frequency pure tone audiometry for the HHL subgroup (n = 12), subjects with tinnitus (n = 2), and subjects without tinnitus (n = 10); (**d**) speech audiometry in silence for the whole cohort (n = 40), for the HHL subgroup (n = 12), and for the no HHL subgroup (n = 38) (* *p* < 0.05; ** *p* < 0.005; **** *p* < 0.0001) (ns = non significant).

Seventeen patients had a threshold better than 20 dB at four frequencies (500, 1000, 2000, 4000 Hz) on both sides. Averaging the PTA thresholds of both ears, the global PTA threshold was 18.3 dB ± 4.6 [5.1; 27.8] for the cohort (Figure 1a). The interaural difference was 3.9 dB ± 4.2 [0; 17.3]. For the younger subgroup, the global PTA threshold was 12.0 dB ± 4.8 [5; 19.2], with an interaural difference of 2.2 dB ± 1.7 [0; 5] (Figure 1b). For the HHL subgroup, the global PTA threshold was 13.0 dB ± 3.9 [5; 17.5], with an interaural difference of 1.7 dB ± 1.7 [0; 5.9] (Figure 1c).

All patients had normal speech intelligibility in quiet situations, with a mean SRT of 22 dB ± 5.8 [10; 30] for the cohort, 19 dB ± 5.3 [12; 30] for the younger subgroup, 24.3 dB ± 4.8 [15; 30] for the non-HHL subgroup, and 16.7 ± 4.2 [10; 23] for the HHL subgroup (Figure 1d). The mean SRT of the HHL subgroup was significantly different from the mean SRT of the total cohort or the non-HHL subgroup (respectively, *p* = 0.005 or *p* < 0.0001) but not from the younger group.

In REM tests on 30 ears, HAs amplified the signal by 5.1 ± 3.4 dB on average, as measured by the REIG on both sides for the cohort, 4.9 ± 4.4 dB for the younger patients and 2.8 ± 1.1 dB for the HHL subgroup (Table 1). The minimum and maximum REIG were 1.3 ± 1.9 dB at 1.5 kHz and 9.8 ± 6.4 dB at 6 kHz for the cohort; 1.3 ± 2.1 dB at 1.5 kHz and 7.3 ± 7.3 dB at 6 kHz for the younger subgroup; and 1.4 ± 1.3 dB at 4 kHz and 5.6 ± 5 dB at 6 kHz for the HHL subgroup. Amplification concerned only frequencies above 1 kHz to conserve the natural amplification of low frequencies and to optimize conversational speech frequencies.

The average daily time of use of HAs was 9.9 ± 3.1 [4.3; 15.6] h. No patient stopped using the HA after 6 months.

**Figure 2:**
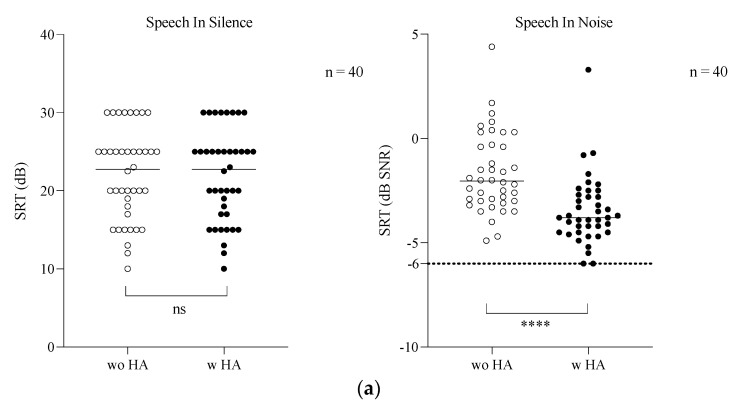

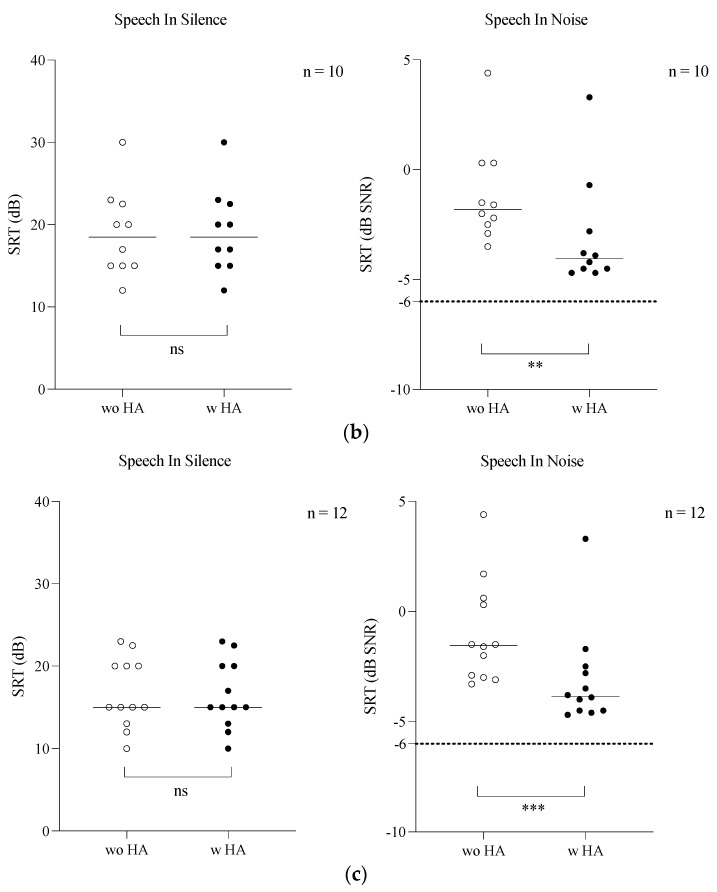
Results for speech in silence and in noise with and without hearing aids (HAs) (**a**) for the cohort (n = 40; **** *p* < 0.0001); (**b**) for the subjects under 45 years old (n = 10; ** *p* < 0.01); and (**c**) for the subjects with HHL (n = 12; *** *p* < 0.001). (w HA = with HA) (wo HA = without HA). SRT in dB SNR is the signal-to-noise ratio (SNR) to reach the speech recognition threshold of 50% (SRT) (ns = non significant).

In quiet situations, there was no difference with (22 ± 5.8 dB) and without (21.9 ± 5.8 dB) HAs in speech intelligibility for the cohort (*p* = 0.58). But HHL patients had a significantly better result in speech audiograms in silence (mean SRT = 16.71 dB) (*p* < 0.0001) compared to non-HHL patients (mean SRT = 24.25 dB). Conversely, FraMatrix results improved significantly (SRT improvement 1.8 dB SNR) from a −1.7 ± 1.9 dB SNR without HAs to a −3.5 ± 1.6 dB SNR with HAs (*p* < 0.001) (Figure 2a). For the younger subgroup, the results were similar, with a significant improvement in the FraMatrix results (SRT improvement of 2.0 dB SNR) from a −1.1 ± 2.3 dB SNR without HAs to a −3.1 ± 2.5 dB SNR with HAs (*p* = 0.002) (Figure 2b). For the HHL subgroup, the results improved significantly on the FraMatrix (SRT improvement of 2.1 dB SNR), from a −1 ± 2.3 dB SNR without HAs to a −3.1 ± 2.1 dB SNR with HAs (*p* = 0.0005) (Figure 2c).

Thirty patients (75%) completed the QOL survey. The APHAB global score significantly improved with HAs (*p* < 0.001). Three subscales (ease of communication, background noise, and reverberation) improved significantly (*p* < 0.001) but averseness was significantly worse with HAs (*p* < 0.05) (Table 1). An improvement of over 20% in the APHAB score was observed in 77% of cases. There was no correlation between APHAB score improvement or FraMatrix results and age, tinnitus severity, or gender.

**Figure 3:**
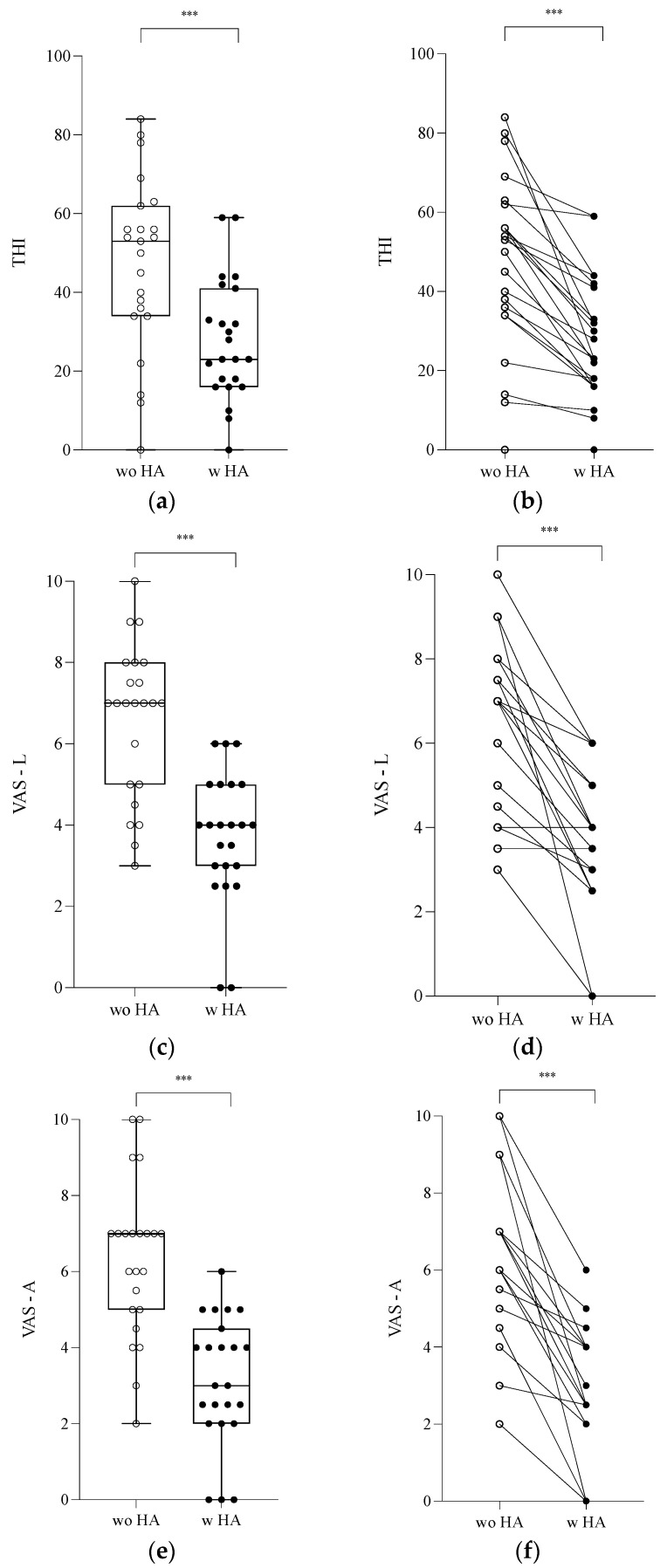
Tinnitus evaluation with and without hearing aids (HAs) (n = 23). Data are represented by whisker box plots (**left**) and individually for each patient (**right**). (**a**,**b**) THI; (**c**,**d**) VAS-L; (**e**,**f**) VAS-A. *** *p* < 0.005, difference between with and without HAs (w HA = with HA) (wo HA = without HA) (THI = Tinnitus Handicap Inventory) (VAS = visual analog scale) (L = loudness) (A = annoyance).

Twenty-three (58%) patients with tinnitus (11 unilateral [48%] and 12 bilateral [52%] cases) were significantly older than patients without tinnitus (56.4 vs. 47.8 years, *p* < 0.05). There was no difference between patients with and without tinnitus in terms of the daily time of use of HAs in the whole cohort (9.2 vs. 10.8 h per day, *p* = 0.15) (Table 1), in the younger subgroup (11.1 vs. 11.4 h per day, *p* = 0.99), or in the HHL subgroup (11.6 vs. 10.8 h per day, *p* = 0.99) (Table 1). The THI score decreased significantly with HAs (47.4 vs. 27.7, *p* < 0.001), as did VAS-L and VAS-A scores (6.6 vs. 3.7 and 6.3 vs. 3.2, respectively; *p* < 0.001) (Figure 3).

There were no between-group differences in the APHAB global scale and subscale results with or without tinnitus, except for ease of communication in the whole cohort and in the younger subgroup (Table 1).

## 4. Discussion

In patients with speech-in-noise difficulties despite normal hearing function in quiet situations, so-called HHL, HAs significantly improved hearing in noise and decreased the impact of tinnitus.

### 4.1. Deafness and Tinnitus

Patients’ difficulties were revealed by testing in a noisy environment in which the coding of suprathreshold sounds and speech is affected. Similar results were observed in patients under 45 (n = 29) while attempting to exclude ARHL cases. It can be assumed that either presbycusis occurs before age 60 or the mechanisms involved do not correspond to those described in presbycusis. The former hypothesis has been highlighted by Yang et al., who reported that ARHL may begin around age 40 in males by affecting high frequencies [28]. Moreover, in a mouse model of ARHL, Parthasarathy et al. showed that the loss of synapses occurs before hearing deterioration [29]. Thus, one can argue that this study explored the very early sign of ARHL, as in the mouse model.

Among our patients, 58% presented with tinnitus, a higher percentage than in the general population [30] (Bhatt et al., 2016), thus reinforcing the hypothesis of a possible common mechanism (cochlear synaptopathy) for HHL and tinnitus [12]. Moreover, Schaette et al. reported that patients with tinnitus had higher thresholds for high standard frequencies (3 to 8 kHz) [12], as observed in 22/23 patients in this cohort.

Animal studies suggest a link between IHC deafferentation and tinnitus, for which several mechanisms have been proposed. The excessive release of glutamate, following sound overexposure or ischemia, can cause excitotoxicity, leading to destruction of the endings of auditory nerve fibers, which innervate IHCs [31]. Tinnitus may be the result of compensatory plasticity, wherein the synaptic gain in auditory central circuits is increased when neural signals from the periphery are attenuated. Studies support the long-standing hypothesis of reduced afferent outflow from a damaged cochlea. Both diminished input and higher auditory centers increase central gain, which may underlie tinnitus [12]. Tinnitus may therefore reflect an early stage of deafferentation mechanisms underlying HHL.

CAPD is well studied in children but less so in adults, and this is even more true for hearing aid rehabilitation in patients with CAPD. In adults, few case report studies have published hearing aid outcomes in CAPD [32]. An exception is the study by Roup et al. [19], who reported a high prevalence of CAPD criteria in a cohort of patients with hearing difficulties in noisy environments. The results of this hearing aid trial in adults with these difficulties revealed significant improvements in hearing handicaps, self-perceived auditory processing difficulties, and speech-in-noise performance [19].

### 4.2. Choice of Hearing Aids

The Phonak^®^ Audeo M-90 (R or RT) has high sound quality and the following advantages: directional microphones, sound reducers, and low background noise speaker. In our study, no patient stopped using the device during the 6 months of follow-up.

The minimum chip background noise of all HAs available at the time of the study was for the Audeo M-90 (R or RT) (less than 29 dB SPL, Phonak data). None of the patients were annoyed by the noise and most of them did not hear it.

Altogether, these findings explain why the patients used the HAs on average 10 h per day, a duration comparable to the 10.01 h per day reported by Pasta et al. for 15,905 real-world users of HAs with classical hearing loss in quiet situations [33].

### 4.3. Hearing Aid Parameters and In Vivo Input

The HA used an automatic noise sound reducer, which allowed for a sharper amplification of sounds of interest and avoided a saturation of high-spontaneous-rate ANFs that encode low-intensity sound. But the open-fit coupling used made the filtering of background noise more difficult. Most background noise is supposed to reach the tympanic membrane and prevents the sound reducer from playing its role. The audiologist turned on the automatic directional microphone to amplify target noises (i.e., sound of speech) and to reduce background noise.

The REM strategy is critical for the fitting of HAs on “normal” hearing listeners, since the hearing aid fitting algorithm used in hearing aid software is based on the HL. The aim is to amplify sounds in a noisy environment without blocking the external ear canal and thus degrading auditory performance in a quiet environment.

The REIG was minimal and ranged from 1.3 dB at 1.5 kHz to 9.8 dB at 6 kHz. The gain was lower than that usually used for HAs in “classical” hearing loss, where it is related to the level of hearing loss. We noticed that the amplification of the signal by the HA was significantly lower for patients with HHL (2.8 ± 1.1 dB). One hypothesis could be that the medium-spontaneous-rate ANFs compensate for the missing low-spontaneous-rate ANFs and are stimulated by sounds of lower intensity.

### 4.4. Benefits of Hearing Aids for Hearing Function and Tinnitus

The ribbon synapse converts the membrane potential of IHCs into spikes in the ANFs [34]. But spikes also occur spontaneously in ANFs contacting the same IHC [35]. Thus, the literature classifies ANFs by high, medium, and low spontaneous rates (SRs) of excitation, which is inversely related to their threshold sensitivity [36]. Low-SR ANFs constitute a subgroup of neurons important for suprathreshold processing. We therefore hypothesized that because low-SR ANFs are the most vulnerable [37], they disappear before medium-SR ANFs. The latter can probably compensate for a long time before the high-SR ANFs are impacted, affecting the sound encoding and sharing a degenerative impact on audiograms in a quiet environment. This hypothesis could be highlighted by the fact that SRT in quiet environments is significantly lower for a patient with HHL (16.7 ± 4 dB) compared to the younger group and the cohort. This could be explained by a higher expression of high- and medium-SR ANFs. Moreover, when sound intensity increases, ANF activity increases and the recruitment of other types of ANF is activated [38]. This recruitment could explain the effect of HAs in our patients, but its mechanism has never been reported in the literature. Another hypothesis for the efficacy of HAs reported in this study is a modification of the ANF phenotype in a manner like that occurring in regeneration. Nouvian et al. reported that after inner ear insult, some ANFs regenerate their extensions and recreate functional synapses with a change in phenotype [39].

HAs also effectively suppressed tinnitus in patients regardless of the THI, VAS-L, and VAS-A scores, which were significantly improved (*p* < 0.001). This efficacy of HAs seems to be explained by two mechanisms. The first mechanism is the masking of tinnitus by the new perception of surrounding noises, in other words, by enriching the soundscape. The amplification of soft sounds allows for masking by ambient noise. The second is the stimulation by higher intensities targeting the frequency band of interest. This leads to fiber saturation and reduces central gain by allowing for residual inhibition [40].

### 4.5. Limitation and Strength of the Study

The study only included 40 patients. The sample size is small and concentrated in middle-aged and elderly people. This may affect the generalizability of the results. We are working on future expansion of the sample size, especially for younger patients with HHL.

The study lasted only 6 months, so it may be difficult to evaluate the long-term benefits. Also, a long-term follow-up should be carried out in the future to explore the stability of the effect.

This is the first study reporting hearing aid benefits in hidden hearing loss patients. We also look at the benefit in terms of tinnitus improvement in this population using precise evaluation questionnaires. We also used tests in noise and REM measurements to precisely report the fitting of hearing aids and patient improvement.

The otoacoustic emissions (OAEs), especially distortion-product OAEs (DPOAEs), could have a potential diagnostic value in the context of HHL. OAEs can provide additional insights into cochlear health and may serve as a complementary tool to identify synaptopathy versus outer hair cell dysfunction. OEAs are expected to be normal in HHL [41].

## 5. Conclusions

We confirm the effect of HAs on intelligibility in noise for patients reporting hearing difficulties in challenging conditions. Moreover, we report for the first time the efficacy of HAs in improving hearing in HHL patients in noisy environments. Despite the small number of patients included and the short follow-up, our study has two main strengths, namely the fitting of the same HA in all patients and the use of REM.

## Figures and Tables

**Table 1:** Principal characteristics of patients without and with tinnitus in the cohort (n = 40), in the subgroup under 45 years old (n = 10), and in the subgroup with hidden hearing loss (n = 12). Results are expressed as a mean value (±standard deviation) and [minimum − maximum]. (APHAB = Abbreviated Profile of Hearing Aid Benefit; EC = ease of communication; BN = background noise; RV = reverberation; AV = averseness; REIG = real-ear insertion gain; PTA = pure tone average; HHL = hidden hearing loss; dB = decibel; SNR = sound-to-noise ratio; SRT = speech recognition threshold.)

	**Whole Cohort**			**Patients with HHL**			**Patients Without HHL**		
	**With Tinnitus**	**Without Tinnitus**	**Total**	**With Tinnitus**	**Without Tinnitus**	**Total**	**With Tinnitus**	**Without Tinnitus**	**Total**
n	23	17	40	2	10	12	7	21	28
Sex	9 w/14 m	11 w/6 m	20 w/20 m	1 w/1 m	7 w/3 m	8 w/4 m	4 w/3 m	8 w/13 m	12 w/16 m
Age (years)	56.4 ± 10.4	47.8 ± 14.1	52.9 ± 12.7	45 ± 8.5	48.3 ± 13.6	47.8 ± 12.6	47.1 ± 15.9	57.7 ± 9.9	55.1 ± 12.3
Daily wearing time (hours)	9.2 ± 3.4	10.8 ± 2.7	9.9 ± 3.1	11.6 ± 4.9	10.8 ± 2.2	11 ± 2.5	10.7 ± 3.5	8.8 ± 3.2	9.4 ± 3.3
PTA (dB)	12.8 ± 5	14.7 ± 3.7	13.6 ± 4.5	9.8 ± 6.7	13.7 ± 3.3	13 ± 3.9	16.2 ± 4	13.1 ± 4.9	13.8 ± 4.8
All frequencies average (dB)	19.1 ± 4.6	16.8 ± 4.9	18.1 ± 4.8	10.7 ± 3.3	14.1 ± 3.5	13.6 ± 3.6	20.4 ± 4.4	19.9 ± 3.8	20 ± 3.9
Speech in silence SRT (dB)	23.5 ± 5.4	19.9 ± 5.8	22 ± 5.8	15 ± 0	17.1 ± 4.6	16.7 ± 4.2	24 ± 4.9	24.3 ± 5	24.3 ± 4.8
Speech in noise SRT (dB SNR)	−1.7 ± 1.2	−2 ± 1	−1.7 ± 1.9	−2.5 ± 0.7	−0.7 ± 2.4	−1 ± 2.3	−2.1 ± 1.9	−1.9 ± 1.6	−2 ± 1.7
REIG 1.5 kHz (dB)	1.2 ± 2.3	1.3 ± 1.6	1.3 ± 1.9	2.3 ± 3.2	1.4 ± 2.1	1.7 ± 2.2	1.3 ± 0.4	0.6 ± 2	0.8 ± 1.6
REIG 2 kHz (dB)	0.9 ± 2.3	6.4 ± 7.8	3.6 ± 5.1	2 ± 2.8	3.4 ± 5.1	3 ± 4.2	12.3 ± 10.3	0.4 ± 2.3	4.3 ± 7.9
REIG 3 kHz (dB)	4.8 ± 6.9	5.9 ± 5.4	5.3 ± 5.9	2.5 ± 2.8	2.6 ± 2	2.6 ± 2	12.5 ± 1.4	5.9 ± 8.4	8.1 ± 7.4
REIG 4 kHz (dB)	5.8 ± 6.2	4.1 ± 4.8	4.9 ± 5.4	0.8 ± 0.4	1.8 ± 1.5	1.4 ± 1.3	8.8 ± 6.7	8.3 ± 6.2	8.4 ± 5.7
REIG 6 kHz (dB)	13 ± 4.6	6.6 ± 6.5	9.8 ± 6.4	9 ± 7.1	3.9 ± 3.7	5.6 ± 5	12 ± 9.2	15 ± 1.8	14 ± 4.6
APHAB EC	21.4 ± 13.2	33.8 ± 18.8	25.9 ± 16.3	9 ± 8.8	38.1 ± 18.2	31.6 ± 20.6	26.3 ± 19.8	22.9 ± 13.1	23.5 ± 14
APHAB BN	60.9 ± 22.1	67 ± 19.4	63.1 ± 21	45.6 ± 6	76.8 ± 6.9	69.8 ± 15.1	50 ± 23.6	62.7 ± 22.6	60.3 ± 22.8
APHAB RV	41.1 ± 19.1	40.6 ± 15.5	40.9 ± 17.6	19.9 ± 13.2	43.6 ± 15.8	38.4 ± 17.9	35.3 ± 15.5	43.6 ± 18.3	42.1 ± 17.8
APHAB AV	21.2 ± 24.3	15 ± 15.9	18.9 ± 21.5	53.9 ± 40.8	17.4 ± 17.6	25.5 ± 26.4	10.8 ± 13.9	17.3 ± 20.3	16.1 ± 19.1
APHAB global	36.1 ± 9.8	39.5 ± 14	37.4 ± 11.4	32.1 ± 6.2	44.6 ± 12.2	41.8 ± 12.5	30.6 ± 12.9	36.6 ± 10.1	35.5 ± 10.6
									
	**Whole Cohort**			**Patients Under 45 Years Old**			**Patients over 45 Years Old**		
	**With Tinnitus**	**Without Tinnitus**	**Total**	**With Tinnitus**	**Without Tinnitus**	**Total**	**With Tinnitus**	**Without Tinnitus**	**Total**
n	23	17	40	3	7	10	20	10	30
Sex	9 w/14 m	11 w/6 m	20 w/20 m	1 w/2 m	4 w/3 m	4 w/5 m	8 w/12 m	7 w/3 m	15 m/15 m
Age (years)	56.4 ± 10.4	47.8 ± 14.1	52.9 ± 12.7	41.7 ± 3.1	33.7 ± 9.7	36.1 ± 8.9	58.9 ± 9.1	57.7 ± 5.3	58.5 ± 7.9
Daily wearing time (hours)	9.2 ± 3.4	10.8 ± 2.7	9.9 ± 3.1	11.1 ± 4.2	11.4 ± 3.6	11.3 ± 3.5	8.9 ± 3.3	10.4 ± 2	9.5 ± 2.9
PTA (dB)	12.8 ± 5	14.7 ± 3.7	13.6 ± 4.5	8.2 ± 5.5	13.7 ± 3.7	12 ± 4.8	13.5 ± 4.6	15.4 ± 3.7	14.1 ± 4.4
All frequencies average (dB)	19.1 ± 4.6	16.8 ± 4.9	18.1 ± 4.8	14.3 ± 3.1	15.2 ± 5.3	14.9 ± 4.6	19.8 ± 4.3	17.9 ± 4.5	19.1 ± 4.4
Speech in silence SRT (dB)	23.5 ± 5.4	19.9 ± 5.8	22 ± 5.8	15.7 ± 1.2	20.4 ± 5.8	19 ± 5.3	24.7 ± 4.8	19.6 ± 6	23 ± 5.7
Speech in noise SRT (dB SNR)	−1.7 ± 1.2	−2 ± 1	−1.7 ± 1.9	−2.7 ± 0.8	−0.5 ± 2.5	−1.1 ± 2.3	−1.9 ± 1.7	−1.9 ± 2.1	−1.9 ± 1.8
REIG at 1.5 kHz (dB)	1.2 ± 2.3	1.3 ± 1.6	1.3 ± 1.9	2.3 ± 3.2	1.8 ± 1.9	1.9 ± 2.1	0.6 ± 2	0.5 ± 0.7	0.6 ± 1.6
REIG at 2 kHz (dB)	0.9 ± 2.3	6.4 ± 7.8	3.6 ± 5.1	2.0 ± 2.8	8.3 ± 8.7	6.2 ± 7.6	0.4 ± 2.3	2.5 ± 3.5	1.1 ± 2.6
REIG at 3 kHz (dB)	4.8 ± 6.9	5.9 ± 5.4	5.3 ± 5.9	4.5 ± 0	4.6 ± 5.9	4.6 ± 4.6	4.9 ± 8.9	8.5 ± 4.2	6.1 ± 7.4
REIG at 4 kHz (dB)	5.8 ± 6.2	4.1 ± 4.8	4.9 ± 5.4	4.0 ± 4.2	4.4 ± 6.2	4.3 ± 5.2	6.6 ± 7.4	3.5 ± 0.7	5.6 ± 6
REIG at 6 kHz (dB)	13 ± 4.6	6.6 ± 6.5	9.8 ± 6.4	9.0 ± 7.1	6.5 ± 8.4	7.3 ± 7.3	15 ± 1.8	6.8 ± 1.8	12.3 ± 4.5
APHAB EC	21.4 ± 13.2	33.8 ± 18.8	25.9 ± 16.3	8.1 ± 10	34.8 ± 17.4	28.1 ± 19.6	23 ± 12.9	32.7 ± 22.3	25.2 ± 15.5
APHAB BN	60.9 ± 22.1	67 ± 19.4	63.1 ± 21	34.3 ± 10	65.2 ± 23.3	57.4 ± 24.6	64 ± 21	69.3 ± 16	65.2 ± 19.8
APHAB RV	41.1 ± 19.1	40.6 ± 15.5	40.9 ± 17.6	21 ± 11.7	42.6 ± 14.3	37.2 ± 16.3	43.5 ± 18.6	38.2 ± 18.2	42.3 ± 18.2
APHAB AV	21.2 ± 24.3	15 ± 15.9	18.9 ± 21.5	21.2 ± 5.4	17.3 ± 18.2	18.3 ± 15.6	21.2 ± 25.8	12.2 ± 14.3	19.1 ± 23.7
APHAB global	36.1 ± 9.8	39.5 ± 14	37.4 ± 11.4	21.1 ± 9.3	40.6 ± 16	35.8 ± 16.7	37.9 ± 8.4	38.1 ± 12.9	38 ± 9.2

## Data Availability

Data are contained within the article.
